# Supplementary material for: Towards routine proteome profiling of FFPE tissue: insights from a 1,220-case pan-cancer study
Source: EMBO J. 2024 Nov 18;44(1):304–29. doi: 10.1038/s44318-024-00289-w (PMC11697351; doi:10.1038/s44318-024-00289-w)
Supplement: Supplementary file 1 — Appendix [file 44318_2024_289_MOESM1_ESM.pdf]

Appendix - Table of contents

Appendix to the EMBO J manuscript "Towards routine proteome profiling of FFPE tissue: insights from a 1,220-case pan-cancer study" by Tüshaus, Eckert & Schliemann *et al.* in 2024.

Appendix Figure	Figure Title	Page
S1	PROCAL spike-in and HeLa quality control runs across the pan-cancer cohort.	1
S2	Comparison to previously published pan-cancer studies using fresh frozen tissue.	2
S3	Quantitative differences of oncogenes and tumor suppressors between cohorts	3
S4	Cancer entity specific fingerprints within the pan-cancer cohort	4

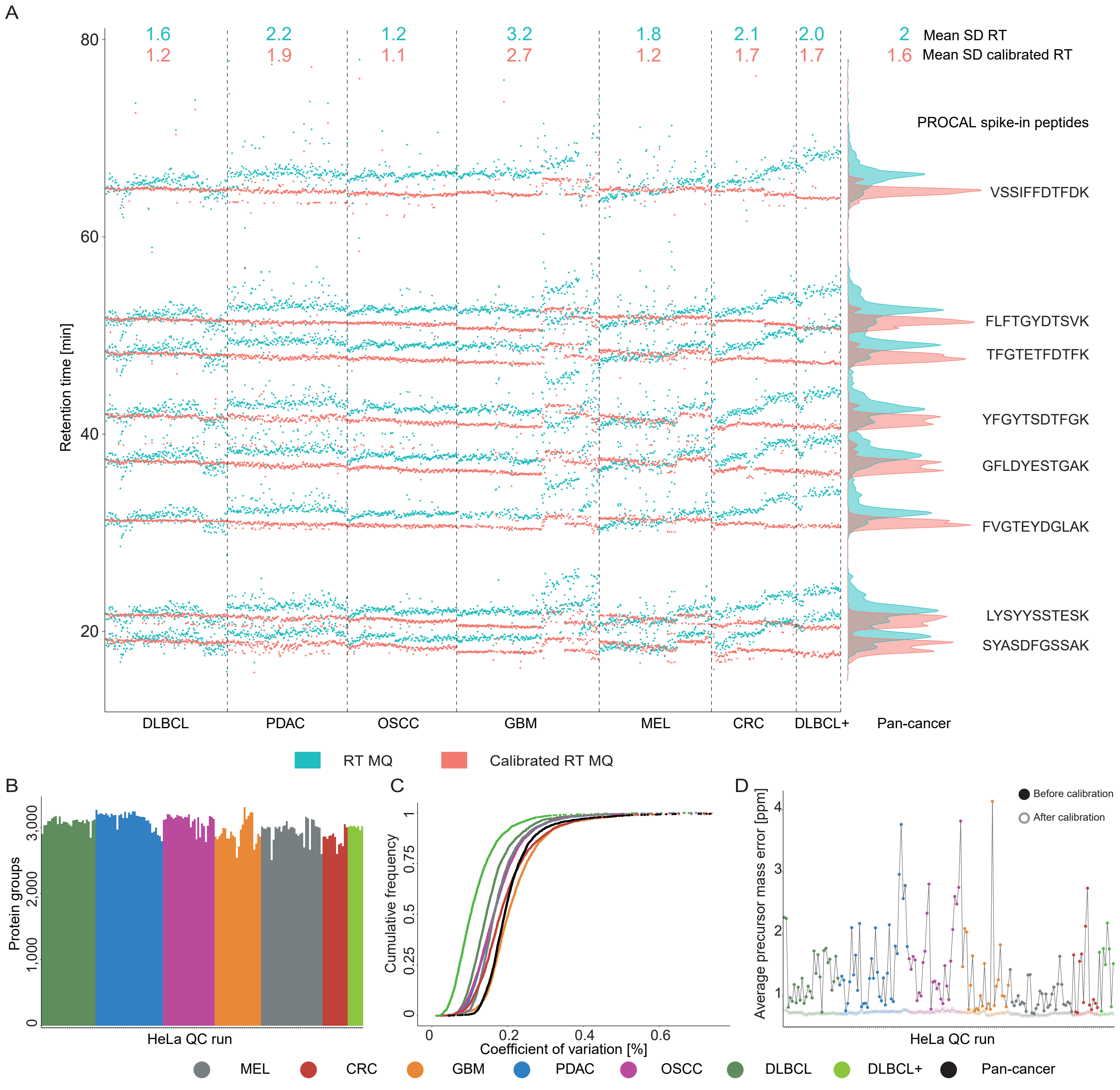

Appendix Figure S1 – PROCAL spike-in and HeLa quality control runs across the pan-cancer cohort.

A) Retention time profiles of synthetic PROCAL peptides spiked into the patient samples as liquid chromatography (LC) quality control before (green) and after (red) retention time recalibration by MaxQuant. The numbers at the top indicate the standard deviation of the retention time for each cohort and across all cohorts (far right) averaged for all PROCAL peptides. **B)** Identified protein groups of HeLa QC samples run over the time span of the pan-cancer cohort project. 300 ng HeLa injections were analyzed using a 44 min gradient with one compensation voltage (-45 V). **C)** Cumulative density plot of the coefficient of variation of LFQ intensities for proteins shared across all HeLa QC runs. Colors refer to HeLa runs that were part of processing the respective cancer entities. **D)** Dot-line plots showing the precursor mass deviation before (filled circles) and after recalibration by MaxQuant (open circles) for all HeLa QC runs in order of acquisition.

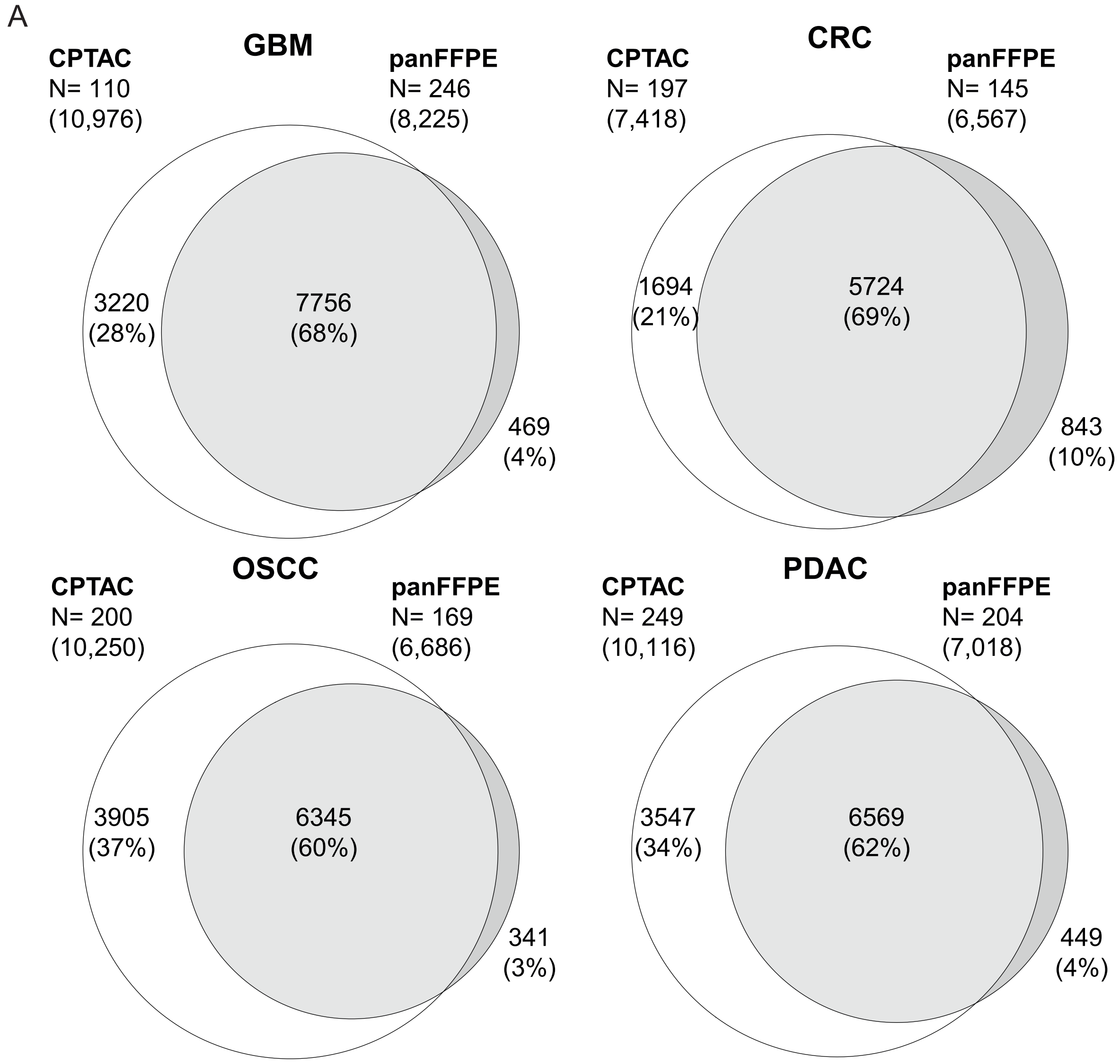

Appendix Figure S2 – Comparison to previously published pan-cancer studies using fresh frozen tissue. A) Venn diagrams illustrating the entity-specific overlap on protein level between our panFFPE study and the CPTAC pan-cancer (fresh-frozen, deep fractionated, TMT-multiplexed) study (Li et al., 2023b; Savage et al., 2024). Preprocessed data published by the CPTAC consortium were. The total number of patients is indicated as well as the number of overall identified proteins.

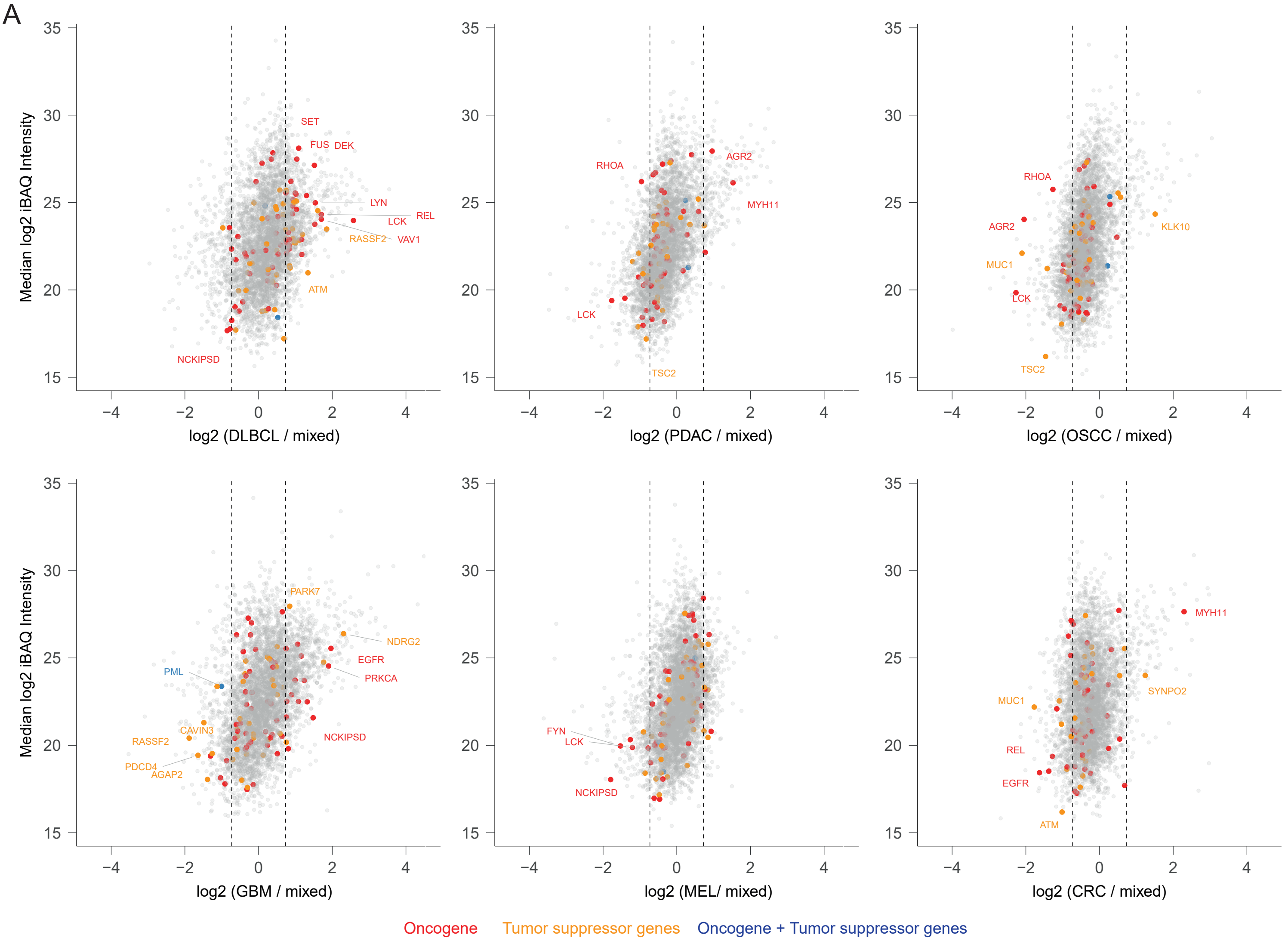

Appendix Figure S3 – Quantitative differences of oncogenes and tumor suppressors between cohorts. A) Scatter plot comparing the expression of all proteins for each entity to the background of all other entities combined. Each dot represents a protein. The log2 fold change of the median protein intensity for the respective entity vs the median protein intensity of all other entities is given on the x-axis and the median log2 iBAQ intensity for the respective cohort is given on the y-axes. The dashed lines represent the fold change cut-off of ±0.73. Proteins annotated as oncogenes and/or tumor suppressors are highlighted in color.

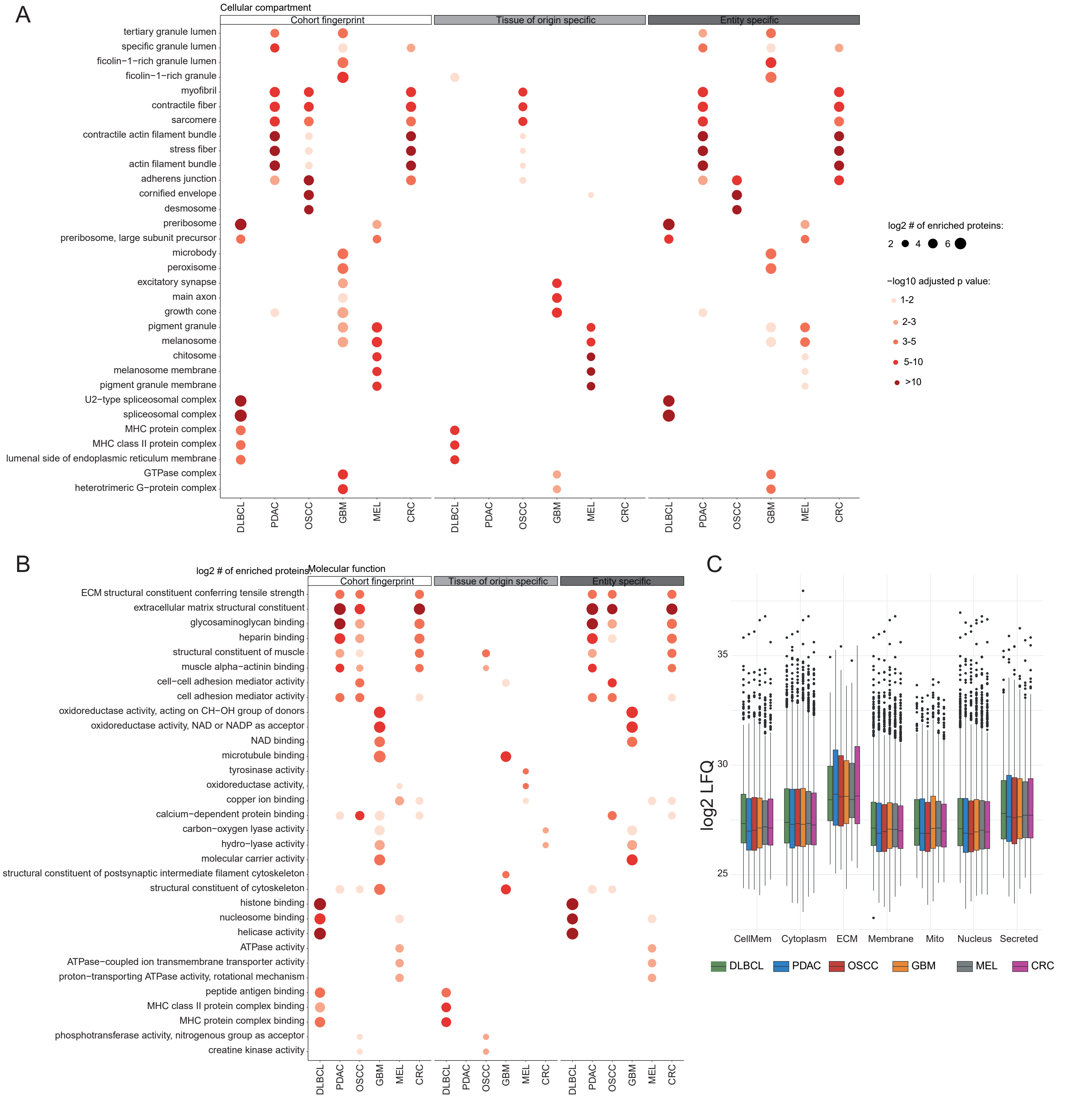

Appendix Figure S4 – Cancer entity specific fingerprints within the pan-cancer cohort. A) GO-term enrichment analysis (cellular compartment) of the cohort fingerprint, tissue of origin and cancer entity specific proteins across all cohorts. The dot size represents the number of enriched proteins for a given GO-term and the colour scale indicates the statistical significance of the enrichment. **B)** Same as A) but for the GO term category “molecular function”. **C)** Boxplot of the log2 LFQ intensities of proteins according to their cellular compartment annotation (Uniprot) for all cohorts.
