# Supplementary material for: Towards routine proteome profiling of FFPE tissue: insights from a 1,220-case pan-cancer study
Source: EMBO J. 2024 Nov 18;44(1):304–29. doi: 10.1038/s44318-024-00289-w (PMC11697351; doi:10.1038/s44318-024-00289-w)
Supplement: Supplementary file 4 — Expanded View Figures [file 44318_2024_289_MOESM4_ESM.pdf]

Expanded View Figures

Figure EV1. Additional information provided by the TIC normalization approach.

(**A**) Three MS1 TIC chromatograms of the same exemplary patient sample. Top: pre-analytical LC-FAIMS-MS run after the first sample preparation with low quality. Middle: pre-analytical LC-FAIMS-MS run after processing the sample a second time. Bottom: final analytical LC-FAIMS-MS/MS run of the reprocessed sample. (**B**) Scatter plot of the log10 sum of the MS1 TIC intensity of pre-analytical LC-FAIMS-MS runs as a function of the date of the first diagnosis as a proxy for the age of the processed FFPE sample. Each dot represents one sample from the melanoma cohort.

 © The Author(s)

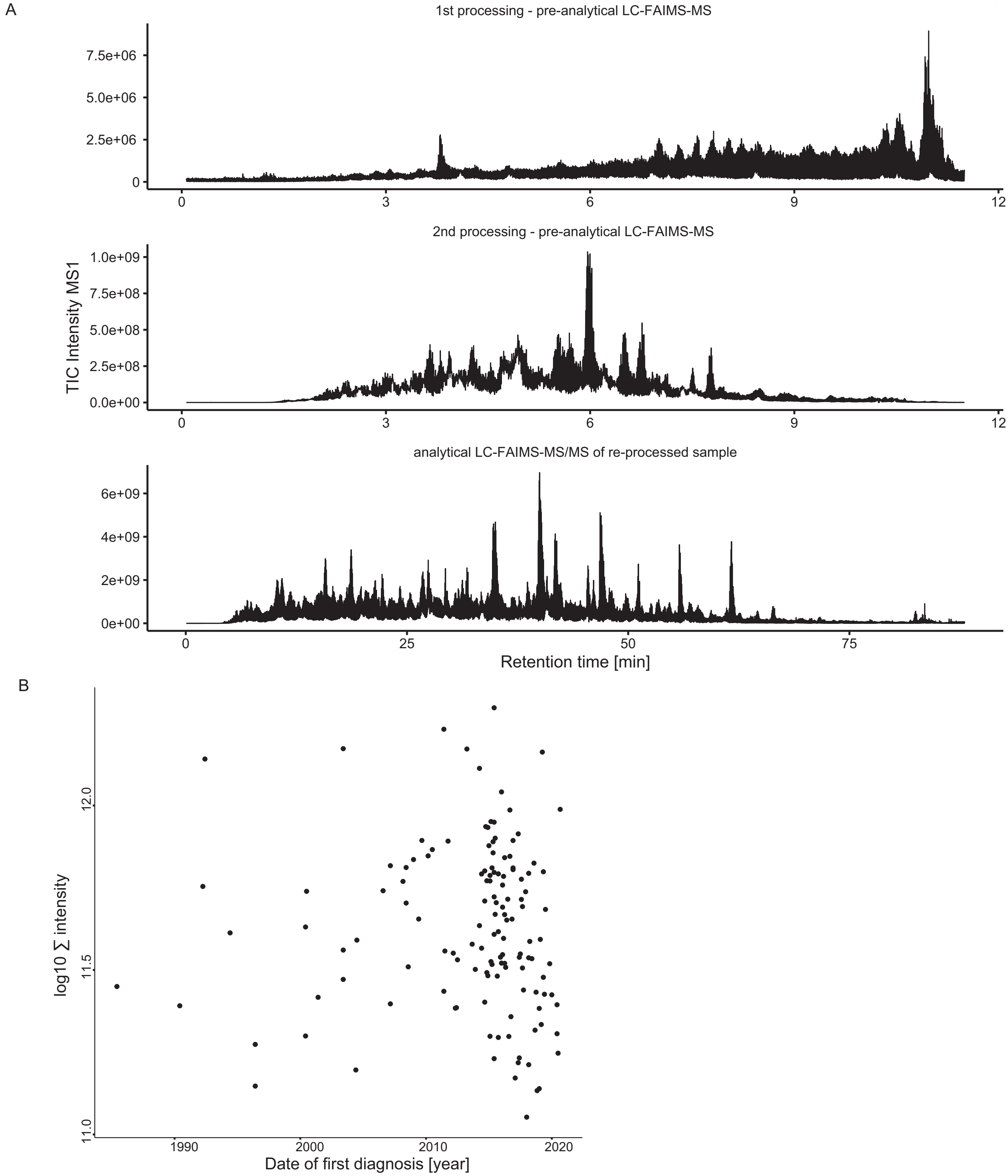
A
1st processing - pre-analytical LC-FAIMS-MS
2nd processing - pre-analytical LC-FAIMS-MS
analytical LC-FAIMS-MS/MS of re-processed sample
TIC Intensity MS1
Retention time [min]
B
log10 ∑ intensity
Date of first diagnosis [year]

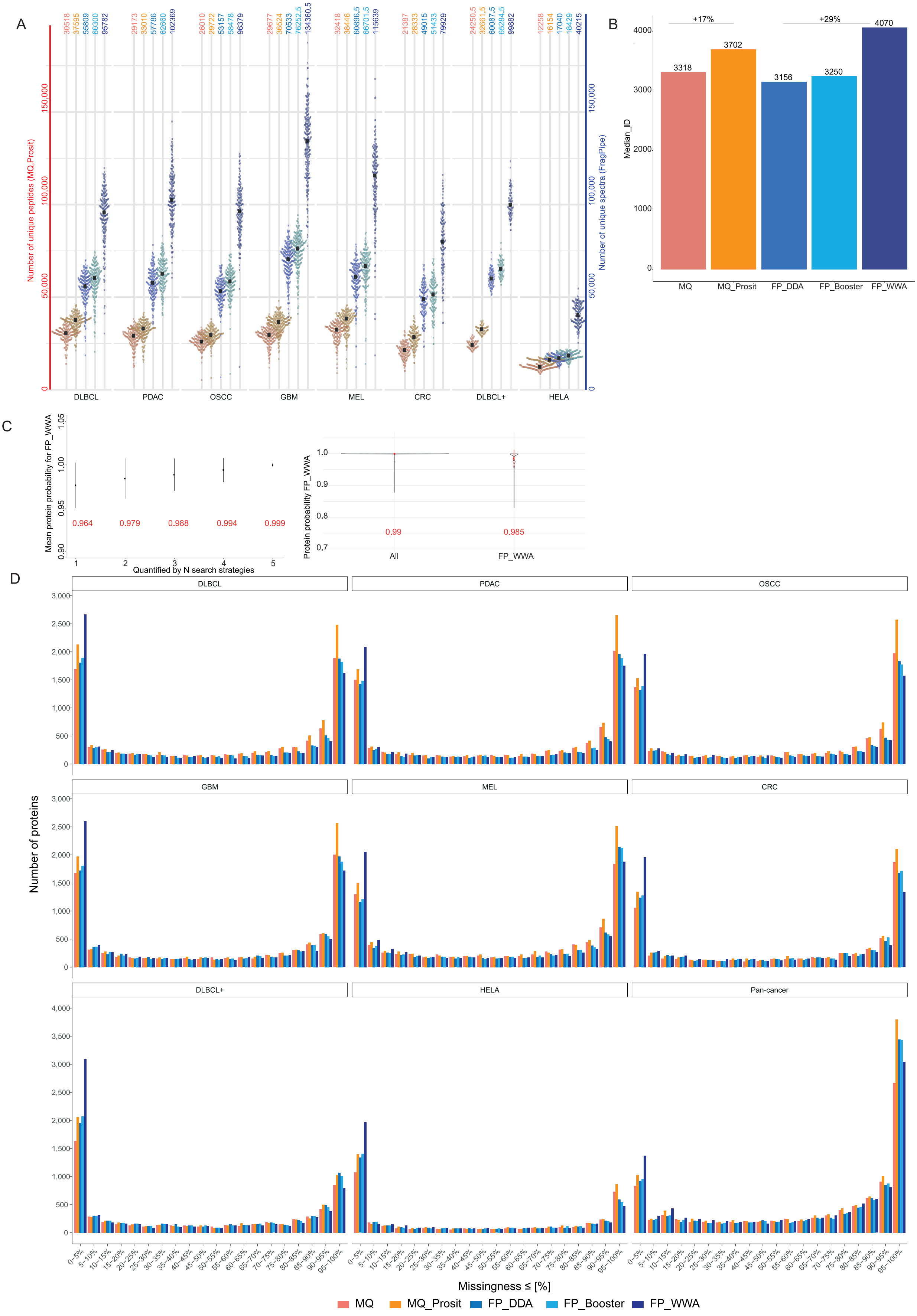
A
Number of unique peptides (MQ,Prosit)
Number of unique spectra (FragPipe)
0
50,000
100,000
150,000
30518 37595 55809 60300 95782
29173 33010 57786 62660 102369
26010 29722 53157 58478 96379
29677 36524 70533 76252.5 134360.5
32418 38446 60896.5 66701.5 115639
21387 28333 49015 51433 79929
24250.5 32661.5 60087.5 65284.5 99882
12258 16154 17040 18429 40215
DLBCL PDAC OSCC GBM MEL CRC DLBCL+ HELA
B
Median_ID
+17%
+29%
3318 3702 3156 3250 4070
MQ MQ_Prosit FP_DDA FP_Booster FP_WWA
C
Mean protein probability for FP_WWA
0.964 0.979 0.988 0.994 0.999
Quantified by N search strategies
Protein probability FP_WWA
0.99 0.985
All FP_WWA
D
DLBCL PDAC OSCC
GBM MEL CRC
DLBCL+ HELA Pan-cancer
Number of proteins
Missingness ≤ [%]
0–5% 5–10% 10–15% 15–20% 20–25% 25–30% 30–35% 35–40% 40–45% 45–50% 50–55% 55–60% 60–65% 65–70% 70–75% 75–80% 80–85% 85–90% 90–95% 95–100%
MQ MQ_Prosit FP_DDA FP_Booster FP_WWA

 © The Author(s)

Figure EV2. Comparison of different search strategies for the analysis of the pan-cancer cohort.

(**A**) Swarm plot indicating the number of unique peptides for the MaxQuant-based searches and the number of unique spectra for the FragPipe-based searches per FFPE tissue sample grouped by entity using different search strategies followed by picked protein group FDR in the following order MaxQuant (red), MaxQuant+Prosit (orange), FragPipe LFQ workflow without MSBooster (blue), FragPipe LFQ workflow with MSBooster (light blue) and FragPipe WWA (dark blue). (**B**) Bar plot showing the median number of quantified proteins per search strategy across all cohorts (HeLa excluded). The gains of post-processing are indicated in percent. (**C**) left: Dot whisker plot showing the mean protein identification probability for FragPipe WWA after picked group FDR for proteins as a function of the number of search strategies the protein was quantified in. 768, 565, 419, 774 and 8560 proteins were quantified by 1, 2, 3, 4 and 5 search engines, respectively. The whiskers represent the standard deviation. Right: Violine plots showing the distribution of the protein identification probability after picked group FDR for proteins that were quantified by all search strategies ($n = 8560$) vs. those quantified by FragPipe WWA but not quantified by all others ($n = 1631$). The red numbers and the red dot indicate the mean values, the whiskers the standard deviation. (**D**) Bar plots showing the number of missing proteins (in bins of 5%) for all five search strategies for all cohorts separately and all cohorts combined.

© The Author(s)

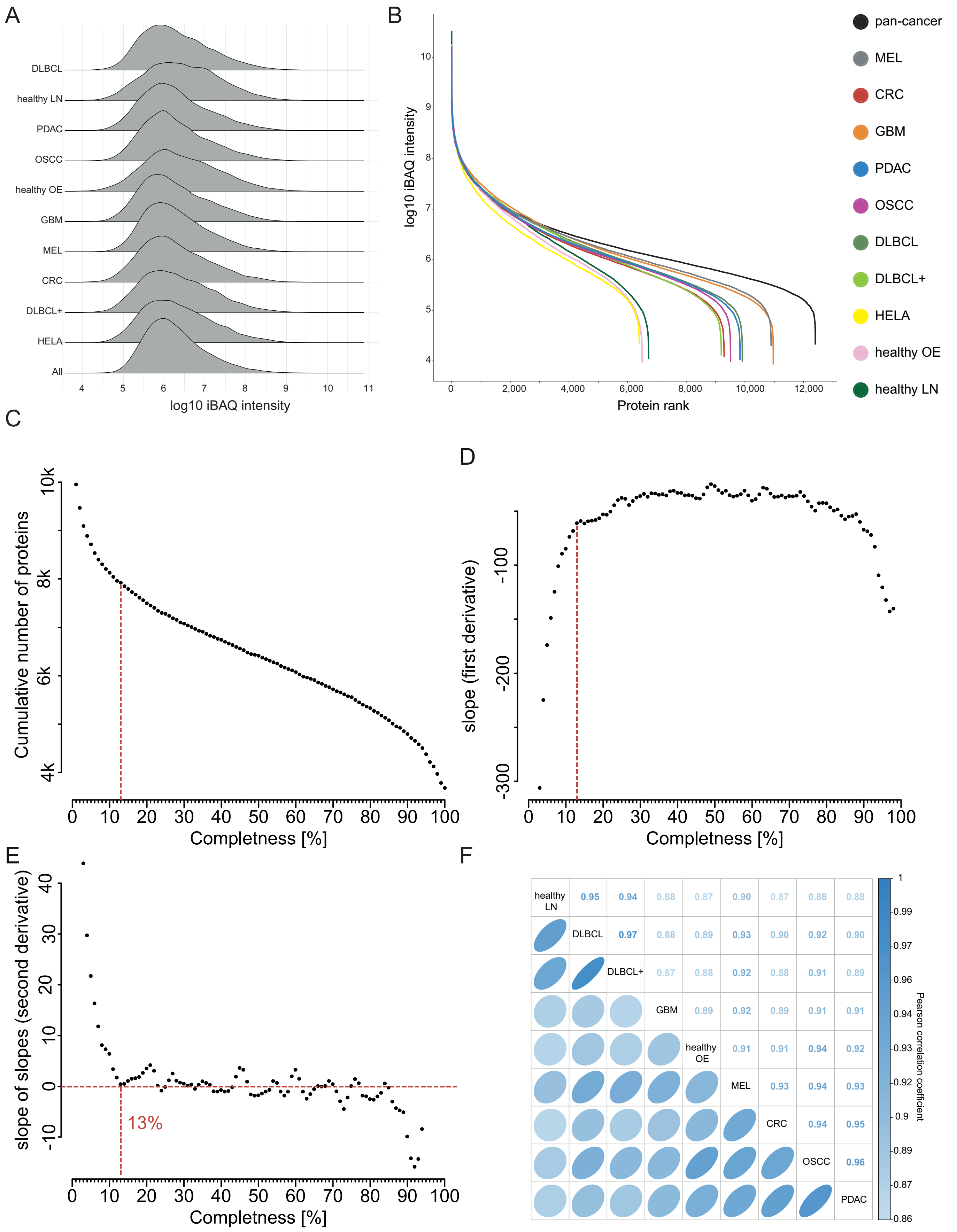
A
DLBCL
healthy LN
PDAC
OSCC
healthy OE
GBM
MEL
CRC
DLBCL+
HELA
All
log10 iBAQ intensity
B
log10 iBAQ intensity
Protein rank
pan-cancer
MEL
CRC
GBM
PDAC
OSCC
DLBCL
DLBCL+
HELA
healthy OE
healthy LN
C
Cumulative number of proteins
Completness [%]
D
slope (first derivative)
Completness [%]
E
slope of slopes (second derivative)
13%
Completness [%]
F
Pearson correlation coefficient

 © The Author(s)

◀ **Figure EV3. Proteomic depth and definition of a completeness cutoff.**

(**A**) Ridge plots showing the distribution of the median log10 iBAQ values for all cohorts separately, HeLa QC samples and all cohorts combined (excluding HeLa samples). (**B**) Abundance rank plot of the median log10 iBAQ intensity of all iBAQ quantified proteins over the corresponding iBAQ Rank for each cohort separately and combined (excluding HeLa samples). (**C**) Dot plot showing the number of quantified proteins across all samples of all cohorts as a function of the completeness. The vertical, dashed line shows the chosen cutoff of 13%. (**D**) The approximated first derivative of the relationship displayed in (**C**). The vertical, dashed line shows the chosen cutoff of 13%. (**E**) The approximated second derivative of the relationship displayed in (**C**). The horizontal line highlights zero, no change in slope. The vertical, dashed line shows the chosen cutoff of 13%. (**F**) Correlation plot between cohorts and heathy tissue samples indicating the Pearson correlation coefficient. Cohorts are sorted by hierarchical clustering using Euclidean distance.

© The Author(s)

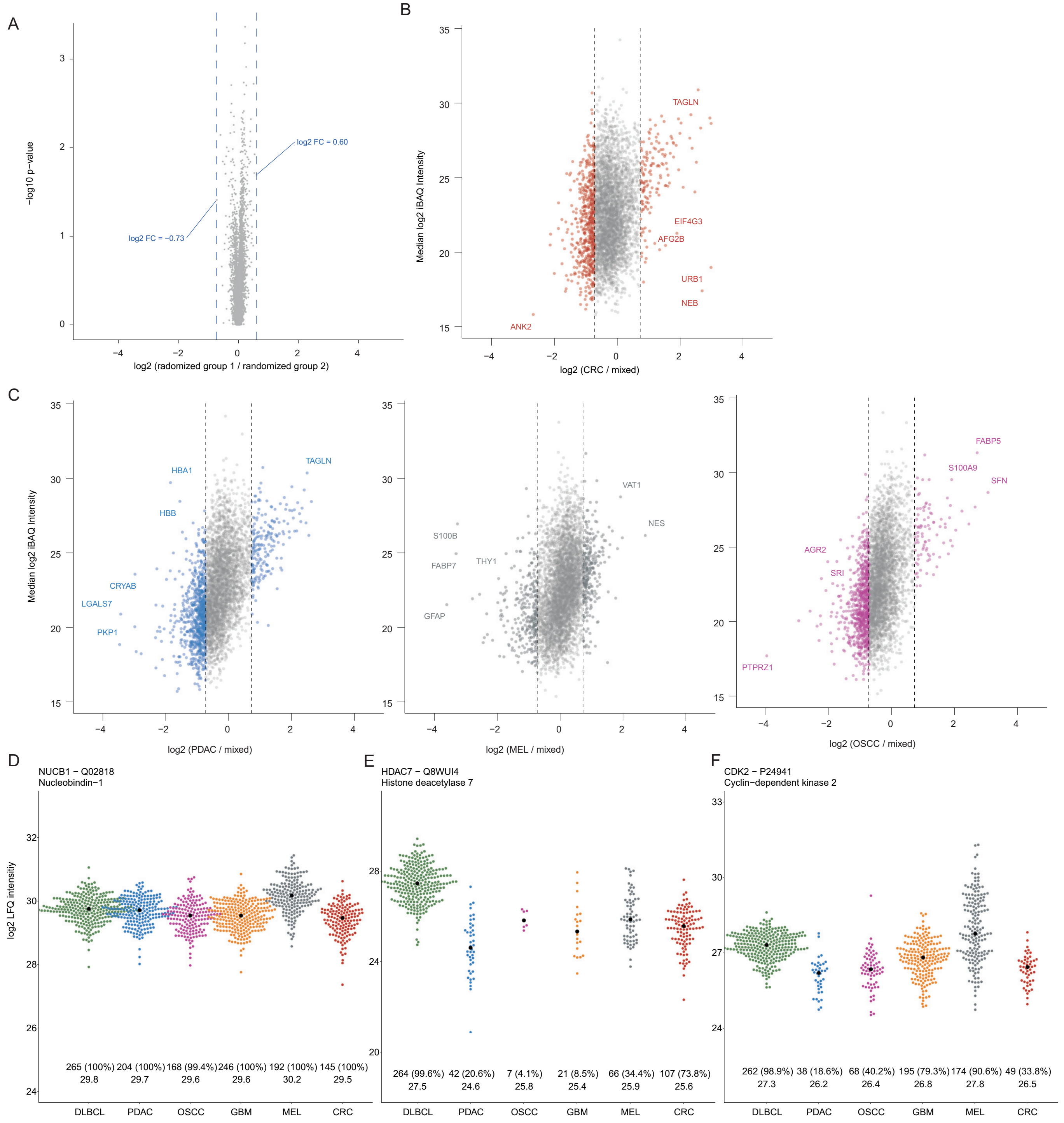
A
−log10 p−value
log2 FC = 0.60
log2 FC = −0.73
log2 (radomized group 1 / randomized group 2)
B
Median log2 iBAQ Intensity
TAGLN
EIF4G3
AFG2B
URB1
NEB
ANK2
log2 (CRC / mixed)
C
HBA1
TAGLN
HBB
CRYAB
LGALS7
PKP1
log2 (PDAC / mixed)
VAT1
NES
S100B
FABP7
THY1
GFAP
log2 (MEL / mixed)
FABP5
S100A9
SFN
AGR2
SRI
PTPRZ1
log2 (OSCC / mixed)
D
NUCB1 − Q02818
Nucleobindin−1
log2 LFQ intensity
265 (100%) 204 (100%) 168 (99.4%) 246 (100%) 192 (100%) 145 (100%)
29.8 29.7 29.6 29.6 30.2 29.5
DLBCL PDAC OSCC GBM MEL CRC
E
HDAC7 − Q8WUI4
Histone deacetylase 7
264 (99.6%) 42 (20.6%) 7 (4.1%) 21 (8.5%) 66 (34.4%) 107 (73.8%)
27.5 24.6 25.8 25.4 25.9 25.6
DLBCL PDAC OSCC GBM MEL CRC
F
CDK2 − P24941
Cyclin−dependent kinase 2
262 (98.9%) 38 (18.6%) 68 (40.2%) 195 (79.3%) 174 (90.6%) 49 (33.8%)
27.3 26.2 26.4 26.8 27.8 26.5
DLBCL PDAC OSCC GBM MEL CRC

© The Author(s)

Figure EV4. Quantitative differences between cohorts.

(**A**) Volcano plot showing the −log10 *P* value of the performed Wilcoxon's Rank test over the log2 fold change for all proteins between group1 and 2 ($n = 609$ patients each). Each patient sample was randomly assigned to one of the two groups, keeping the size of each cohort equal between the two groups. The maximum log2 fold change following this random assignment is indicated (blue). This maximum fold change observed by random chance alone gives insights into the variation in the dataset and allows to define a fold change cutoff for biological relevant comparisons used for further analyses. (**B**) Scatter plot comparing the expression of all proteins for CRC ($n = 145$) to the background of all other entities combined ($n = 1075$) using a Wilcoxon's Rank test. Each dot represents a protein. The log2 fold change of the median protein intensity for the respective entity vs the median protein intensity of all other entities is given on the *x* axes and the median log2 iBAQ intensity for the respective cohort is given on the y-axes. The dashed lines represent the fold change cutoff of ± 0.73 determined from (**A**). (**C**) Left: Same as (**B**) but for PDAC (PDAC: $n = 204$, combined background: $n = 1{,}1016$). Middle: same as (**B**) but for MEL. Right: same as (**B**) but for OSCC ($n = 168$, combined background: $n = 1052$). (**D**) Exemplary protein NUCB1 showing a rather stable degree of variability across all cohorts. The numbers at the bottom indicate the number of samples the protein was quantified in per cohort, the corresponding percentage and the median LFQ intensity. (**E**) Exemplary proteins HDAC7, druggable by small molecule inhibitors, enriched in DLBCL. (**F**) In contrast to NUCB1 in D) CDK2 exhibiting a higher degree of variability in MEL compared to other cohorts.

© The Author(s)

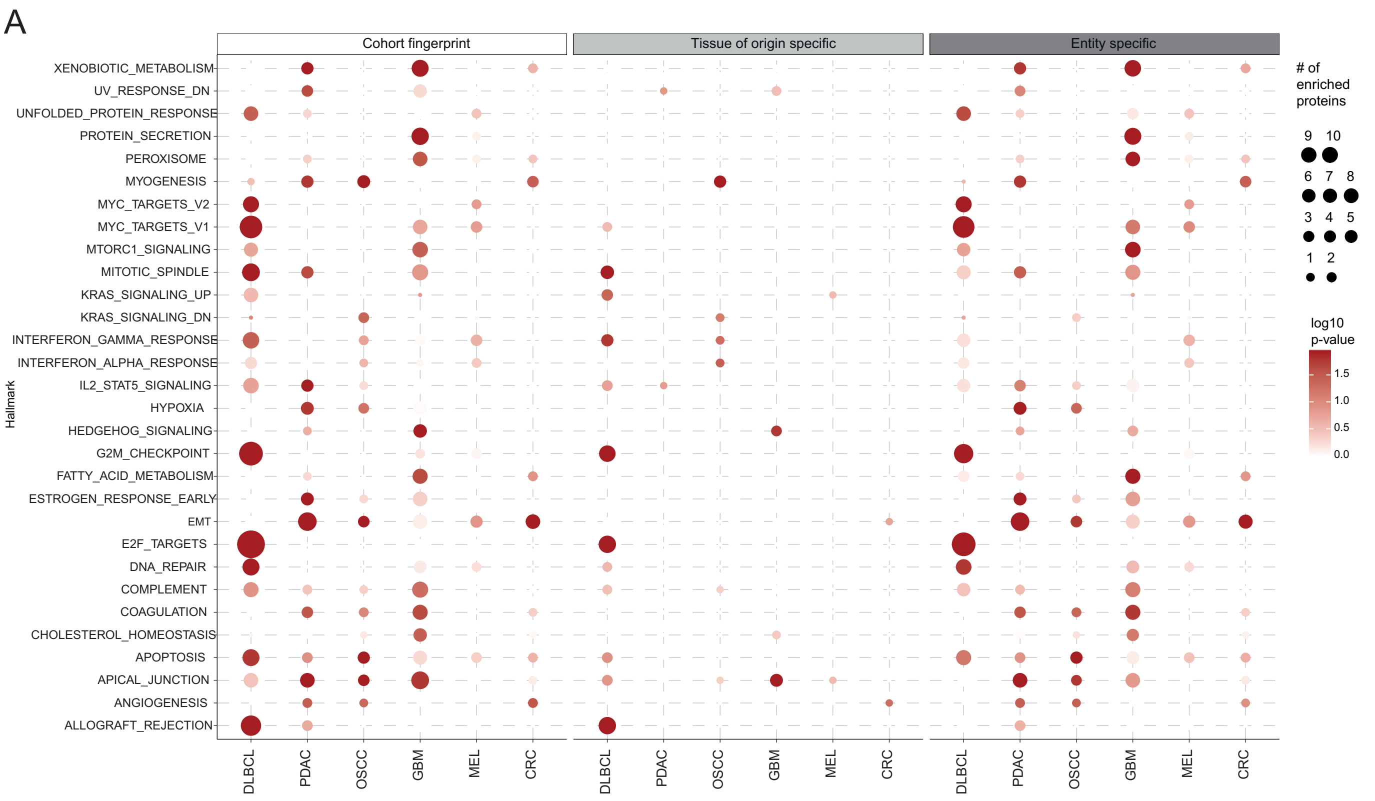

Figure EV5. Hallmark of cancer enrichment analyses and comparison to healthy tissue.

(**A**) Hallmark of cancer overrepresentation analysis based on a chi-squared contingency table test using the hallmark annotation database as background (MSigDB; Liberzon et al, 2015) for the cohort fingerprint, tissue of origin and cancer entity-specific proteins across all cohorts. The dot size represents the number of enriched proteins for the given Hallmark and the color scale indicates the statistical significance of the enrichment.

 © The Author(s)
